# Enhanced Cyclability of Cr_8_O_21_ Cathode for PEO-Based All-Solid-State Lithium-Ion Batteries by Atomic Layer Deposition of Al_2_O_3_

**DOI:** 10.3390/ma14185380

**Published:** 2021-09-17

**Authors:** Haichang Zhang, Zhibin Xu, Bin Shi, Fei Ding, Xingjiang Liu, Hongzhao Wu, Chunsheng Shi, Naiqin Zhao

**Affiliations:** 1 Tianjin Key Laboratory of Composite and Functional Materials, School of Materials Science and Engineering, Tianjin University, Tianjin 300350, China; hchzhang@tju.edu.cn (H.Z.); nqzhao@tju.edu.cn (N.Z.); 2 Science and Technology on Power Sources Laboratory, Tianjin Institute of Power Sources, Tianjin 300384, China; beyond_x@163.com (Z.X.); daiye101@mail.nankai.edu.cn (B.S.); xjliu@nklps.org (X.L.); 3 School of Automotive Engineering, Tianjin Vocational Institute, Tianjin 300410, China; wuhongzhao9059@163.com

**Keywords:** Cr_8_O_21_ cathode material, atomic layer deposition, all-solid-state lithium-ion batteries, Al_2_O_3_ coating

## Abstract

Cr_8_O_21_ can be used as the cathode material in all-solid-state batteries with high energy density due to its high reversible specific capacity and high potential plateau. However, the strong oxidation of Cr_8_O_21_ leads to poor compatibility with polymer-based solid electrolytes. Herein, to improve the cycle performance of the battery, Al_2_O_3_ atomic layer deposition (ALD) coating is applied on Cr_8_O_21_ cathodes to modify the interface between the electrode and the electrolyte. X-ray photoelectron spectroscopy, scanning electron microscope, transmission electron microscope, and Fourier transform infrared spectroscopy, etc., are used to estimate the morphology of the ALD coating and the interface reaction mechanism. The electrochemical properties of the Cr_8_O_21_ cathodes are investigated. The results show that the uniform and dense Al_2_O_3_ layer not only prevents the polyethylene oxide from oxidization but also enhances the lithium-ion transport. The 12-ALD-cycle-coated electrode with approximately 4 nm Al_2_O_3_ layer displays the optimal cycling performance, which delivers a high capacity of 260 mAh g^−1^ for the 125th cycle at 0.1C with a discharge-specific energy of 630 Wh kg^−1^.

## 1. Introduction

Lithium-ion batteries (LIB) based on inorganic transition-metal oxide cathodes, graphitic-carbon anodes, and organic-liquid electrolytes [1] are widely applied in many fields, including portable electronic devices, power tools, electric vehicles (EV), and large-scale energy storage stations due to their high energy density, long cycle life, and low self-discharge rate [2]. However, the commercialized LIBs still cannot meet the increasing demand for energy storage devices with long cycle life and high energy density for EVs with longer cruising distances.

The energy density of lithium-ion batteries mainly depends on the cathode materials. The state-of-the-art LIB cathodes include Li transition-metal oxides and Li transition-metal phosphates [3], which show a capacity of less than 250 mAh g^−1^. However, the energy density above 300 Wh kg^−1^ is desired to increase the driving range and to decrease the cost [4]. To achieve this target, the specific capacity of the cathode material should reach 500 mAh g^−1^. In recent years, Li-free cathodes with high energy density such as S, FeF_2_, FeS_2_, and transition-metal oxides have attracted renewed attention owing to their high theoretical capacity [4,5,6,7,8]. Chromium oxides with high-valence have also been considered as promising cathode materials for lithium-ion batteries due to their ultrahigh theoretical capacity. For example, octachromium henicosaoxide (Cr_8_O_21_) possesses a theoretical capacity of 642 mAh g^−1^ when 18 lithium ions insert into the Cr_8_O_21_ structure totally. Further researches show that Cr_8_O_21_ has an initial capacity of 395 mAh g^−1^ and a reversible capacity of 260 mAh g^−1^ [9,10]. Therefore, the use of oxides with high specific capacity as cathode materials for LIBs is a promising choice to improve the energy density and to achieve low cost. Li-free cathode materials show the potential to provide energy densities higher than traditional Li-ion batteries while coupled with Li metal anodes due to the advantages of high specific capacity (3800 mAh g^−1^) and low reduction potential (−3.04 V vs. the standard hydrogen electrode) [11,12,13,14]. While in a conventional Li-ion battery system, the growth of Li dendrites may induce the internal short circuits that may lead to fire, explosion, and uncontrolled electrolyte decomposition. The use of solid-state electrolytes to construct all-solid-state (ASS) batteries is conducive to circumvent these issues [15,16,17]. In addition, the solid electrolyte can be made extremely thin so that it can enhance the energy density of the battery [15,18]. Therefore, the ASS battery consisted of high-capacity metal oxide cathode and lithium metal anode is considered as one of the most promising next-generation battery systems.

Solid electrolytes are usually classified as inorganic solid electrolytes and polymer electrolytes. Polymer electrolytes have attracted much interest owing to the advantages of enhanced resistance to volume changes of the electrodes during the charge/discharge processes, improved safety features, excellent flexibility, and processability [19]. However, the possible chemical incompatibility between oxide cathode materials and polymer electrolytes should be taken into account. Polyethylene oxide (PEO) is the most promising candidate as a solid solvent for lithium salts owing to the flexible ethylene oxide segments and ether oxygen atoms, which have a strong donor character and thus readily solvate Li^+^ cations. More importantly, it is commercially available in a relatively pure state at a very reasonable cost. Therefore, effective measures should be taken to solve the problems of chemical incompatibility between metal oxides and solid electrolytes to improve the specific energy and the cycling performance of the ASS lithium-ion batteries.

Using low-cost, cobalt-free, and high-performance metal oxides as cathode materials is beneficial to the development of solid-state lithium batteries for practical applications [20]. For example, Zhang et al. [21] and Shim et al. [22] investigated Li-V_2_O_5_ solid-state lithium batteries with a solid polymer electrolyte (PEO and PEG, respectively). These studies have improved the cycling stability, but the number of cycles was still no greater than 50 cycles. In addition, studies on oxides (e.g., Cr_8_O_21_) with higher discharge-specific energy and stronger oxidizing used in solid-state batteries have been rarely reported. In general, there is a side reaction between the cathode material and the solid electrolyte in the solid-state battery [17,23,24], which deteriorates the performance of the battery. When oxides with higher oxidation are selected as cathode materials, the interface problems are more prominent (mainly chemical stability) [17,24]. To solve the interface problem and reduce the interface resistance, interfacial modification is an effective approach [17,23,25]. Meanwhile, the coating layer should have suitable Li ionic conductivity, and chemical and electrochemical stability.

Herein, a new all-solid-state battery was prepared by using Cr_8_O_21_ and lithium metal as the cathode material and the anode material, respectively, and the PEO-LiTFSI as the solid electrolyte. The performance of Cr_8_O_21_ cathode in all-solid-state lithium metal batteries (SSLMBs) was evaluated. The coating of the Al_2_O_3_ layer on the Cr_8_O_21_ electrode by the ALD technique was introduced to prevent the solid electrolytes from strong oxidization of Cr_8_O_21_, which improves the cycle life of the batteries. The capacity retention rate after 125 cycles is 87.4%, and the energy density is up to 630 Wh kg^−1^ (based on Cr_8_O_21_ materials), indicating a promising safe, high energy density secondary battery system.

## 2. Materials and Methods

### 2.1. Preparation of Cr_8_O_21_ Powder

Methods for the preparation of Cr_8_O_21_ powder are similar to those reported previously [9]. Typically, CrO_3_ flakelets (Alfa Aesar, AR, Haverhill, MA, USA; thin sheets with a diameter of approximately 1 cm) were calcinated under oxygen flow (20 mL min^−1^) at 280 °C for 7 h. After cooling to room temperature, the obtained product was ground with agate mortar and sieved through a 300 mesh screen to obtain the Cr_8_O_21_ powder.

### 2.2. Preparation of Cr_8_O_21_ Electrode

To prepare the Cr_8_O_21_ cathode electrodes, the active material (Cr_8_O_21_), Ketjen black (KB, Lion Corporation, Tokyo, Japan), and poly(vinylidene fluoride) (PVdF, Arkema, Colombes, Colombes, France) (8:1:1 by weight) were mixed with an appropriate amount of N-methyl-2-pyrrolidinone (Alfa Aesar, AR, Haverhill, MA, USA) to form a slurry. The obtained slurry was then coated on an Al foil using a doctor blade (100 μm thickness). After drying at 120 °C for 12 h in vacuum, the electrode sheet was cut into round sheets with a diameter of 16 mm for both the test and ALD electrodes.

### 2.3. Preparation of PEO-Based Polymer Electrolyte Membrane

PEO (Mw = 100,000, Sigma-Aldrich, St. Louis, MO, USA) and lithium bis (trifluoromethanesulfonyl imide) (LiTFSI, TCI, Tokyo, Japan) were dissolved in tetrahydrofuran (THF, extra dry, TCI, Tokyo, Japan) with a molar ratio of PEO:LiTFSI = 8:1 under continuously stirring at 60 °C until complete dissolution. Then, 50 wt.% Al_2_O_3_ nano-powder (Aladdin, Shanghai, China) was added to the above solution until mixing uniform. The mixture was subsequently cast into a thin film using a doctor blade (100 μm thickness) and dried at 80 °C for 12 h. The film was further dried in vacuum at 50 °C for another 12 h.

### 2.4. Preparation of ALD-Coated Cr_8_O_21_ Electrode

Al_2_O_3_ was deposited on the Cr_8_O_21_ electrode or on the Si wafer at 140 °C in a Savannah 100 ALD system (Ultratech/Cambridge Nanotech, USA) using trimethylaluminum (TMA, (CH_3_)_3_Al, 98%, STREM Chemicals, Newburyport, MA, USA) and distilled water (H_2_O) as precursors. The ALD details are as follows: (i) Put the electrode into the sample chamber, heat to 140 °C; (ii) Perform H_2_O pulse for 0.2 s, and then react for 45 s, followed by purging for 45 s; (iii) Conduct TMA pulse for 0.1 s, react for 45 s, and then purge for 45 s; (iv) Cool down to room temperature, inflate to atmospheric pressure, and then take out the Cr_8_O_21_ electrodes. Repeat (ii), (iii) steps to achieve the selected number of cycles. In this study, 8, 12, 16, and 20 ALD cycles were selected for the Cr_8_O_21_ electrode, and 100 ALD cycles were selected for Si wafers.

### 2.5. Preparation of All-Solid-State Cell

The PEO (LiTFSI)/THF solution (molar ratio of PEO:LiTFSI = 8:1) was cast on the Cr_8_O_21_ electrodes to obtain composite electrodes with a closely integrated interface, and then the electrodes were dried at 70 °C for 12 h. The obtained Cr_8_O_21_ electrode with PEO-based solid polymer electrolyte membrane and Li metal electrode was assembled together under 20 MPa. Finally, all-solid-state cells were assembled to 2430 coin-type cells in an argon-filled glove box. In accordance with the method described above, an all-solid battery prepared by stainless steel sheet instead of Cr_8_O_21_ electrode was used for investigating the electrochemical stability of the PEO solid electrolyte. In contrast, the conventional organic electrolyte cells were assembled and tested. The Cr_8_O_21_ electrode, Celgard 2320, and lithium metal were used as the cathode, separator, and anode, respectively. A total of 1 M LiPF_6_ (Capchem, Shenzhen, China) in ethylene carbonate (EC, TCI, Tokyo, Japan) and dimethyl carbonate (DMC, TCI, Tokyo, Japan) mixture (volume ratio of EC to DMC was 1:1) was used as the electrolyte. The CR2430 coin-type cells were assembled in an Ar-filled glove box.

### 2.6. Characterizations

Powder X-ray diffraction (XRD, Rigaku M2400, Rigaku Co., Tokyo, Japan) was used to characterize the crystal structure of the Cr_8_O_21_ powder with Cu Kα radiation. Scanning electron microscope (SEM, S-4800, Hitachi Inc., Tokyo, Japan) and transmission electron microscope (TEM, Philips Tecnai G2 F20, FEI Co., Hillsboro, OR, USA) were used to characterize the morphology and microstructure of both the powder and the electrodes. X-ray photoelectron spectroscopy (XPS, Axis Ultra DLD, Shimadzu-Kratos Co. Ltd., Kanagawa, Japan) was used to derive information on the bonding state of the samples. Fourier transform infrared spectroscopy (FTIR, Nicolet 6700, Thermo Fisher, Waltham, MA, USA) was used to characterize the change of PEO-based membrane. Surface profiler (P-l6+, KLA-Tencor, Milpitas, CA, USA) and atomic force microscope (AFM, Agilent AFM5500, Agilent Technologies, Inc., Santa Clara, CA, USA) in tapping mode at 512 Hz were used to calibrate the thickness of 100-ALD-Al_2_O_3_ coated on a silicon plate.

### 2.7. Electrochemical Tests

The electrochemical measurements of all-solid-state cells were performed in a thermostat (Shanghai Bluepard, Shanghai, China) at 60 °C. Before the test, the cells were rest in the thermostat for 30 min. Galvanostatic tests were cycled between 1.5 and 4.2 V by using the LAND-CT2001A battery-testing instrument (Wuhan LAND Electronic Co. Ltd., Wuhan, China). Cyclic voltammetry (CV) and electrochemical impedance spectroscopy (EIS) measurements were conducted on a Solartron 1470 + 1400 electrochemical workstation (AMETEK Inc., Berwyn, PA, USA). The scan potential range of CV measurement was 1.5–4.2 V at a scan rate of 0.1 mV s^−1^ for the Cr_8_O_21_ electrode test and 1.0–5.0 V at a scan rate of 0.01 mV s^−1^ for the electrochemical stability of PEO electrolytes (at 60 °C). The EIS was examined in the frequency range of 10 MHz to 1 Hz with an AC amplitude of 5 mV.

## 3. Results and Discussion

**Figure 1:**
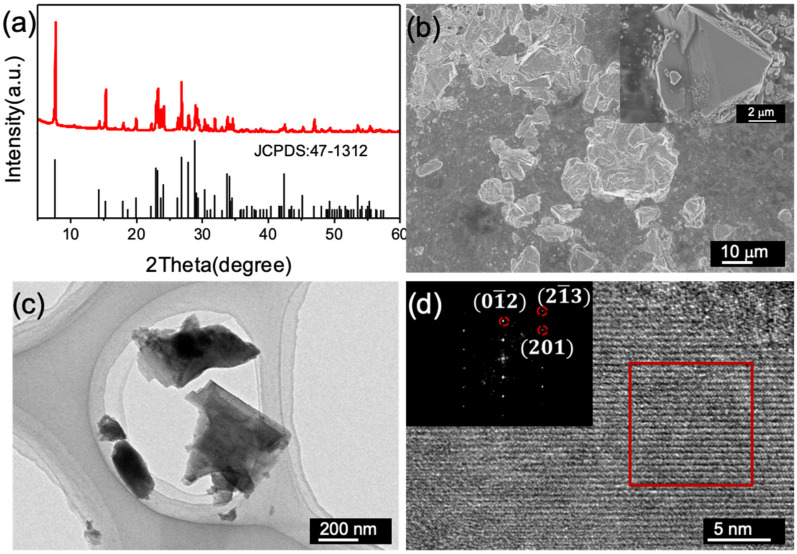
XRD patterns (**a**), SEM image (**b**), TEM image (**c**), and (**d**) HRTEM image and FFT pattern (inset) of the Cr_8_O_21_ powder.

The morphology and the crystal structure of the Cr_8_O_21_ powder were characterized by SEM, TEM, and XRD. Figure 1a shows the XRD pattern of the Cr_8_O_21_ powder, which displays an excellent consistency with the standard card of Cr_8_O_21_ (JCPDS card no. 47-1312). Norby et al. determined that the space group of Cr_8_O_21_ is P1 by using neutron diffraction techniques and verified the oxide state of chromium atoms in Cr_8_O_21_ consisting of two trivalent chromium atoms and six hexavalent chromium atoms [26]. SEM image of the Cr_8_O_21_ powder shown in Figure 1b reveals that the average particle size is approximately 10 μm. These particles exhibit angular surfaces and irregular shapes (Figure 1c). This is mainly due to the fact that the obtained Cr_8_O_21_ powder has been subjected to the process of crushing and grinding. The HRTEM image shown in Figure 1d exhibits a clear lattice pattern, indicating the high crystallinity of the Cr_8_O_21_ particles. The fast Fourier transformation (FFT) pattern (inset in Figure 1d) of the image in red square reveals the triclinic crystal characteristics. The main diffraction spots are assigned to (0$\bar{1}$2), (2$\bar{1}$3), and (201) plane of triclinic Cr_8_O_21_, consistent with the XRD results.

Figure S1a–d present SEM images of the Cr_8_O_21_ electrodes with different ALD cycles (0, 8, 12, 16). The Cr_8_O_21_ particles without ALD coating present a rough surface because of pressing and rubbing during the grinding process. After coating with Al_2_O_3_ by ALD, the surface of the Cr_8_O_21_ particle becomes smooth [27,28], which can be attributed to the high homogeneity of the Al_2_O_3_ ALD coating. Meanwhile, the integrity of the Al_2_O_3_ layer was improved with the increase in ALD-coated cycles. After 8 ALD cycles, the coating cannot completely cover the surface of the Cr_8_O_21_ particles. After 12 ALD cycles and 16 ALD cycles, the Al_2_O_3_ coatings become more homogenous and complete. The Al_2_O_3_-coated Cr_8_O_21_ electrodes were subjected to surface element analysis by EDS to determine the Al element content. The bar graph of Al element content in the coating with different ALD cycles (0, 8, 12, 16) is presented in Figure S2, which indicates that the Al element content increased with increasing ALD coating cycles. When the ALD coating cycle is 8, the Al content is 1.67 wt.%. As the ALD coating cycle number increases to 12 and 16, the Al element contents reach 3.35 and 10.2 wt.%, respectively.

**Figure 2:**
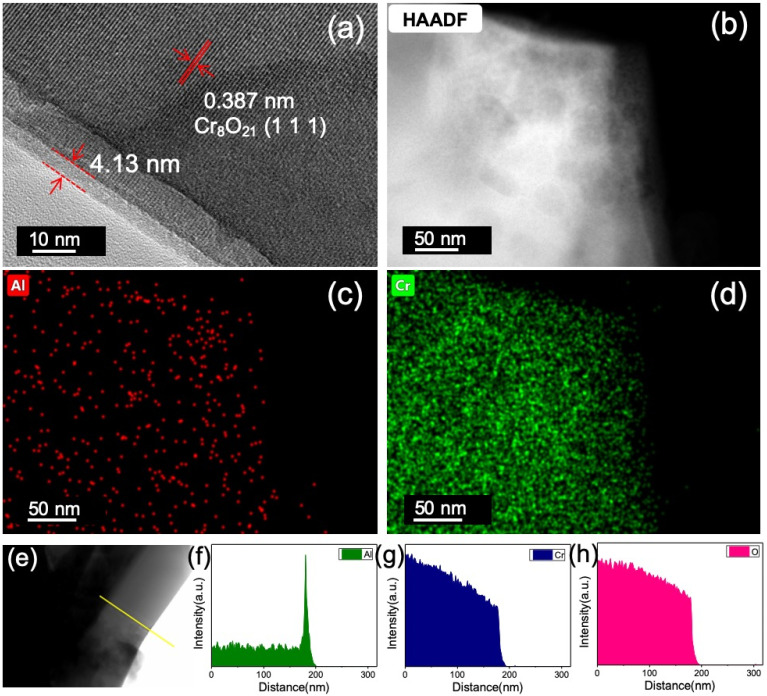
(**a**) TEM image of the Cr_8_O_21_ electrodes with 12 cycles of Al_2_O_3_ ALD, (**b**) TEM image of the EDS mapping region, (**c**,**d**) EDS mapping of Al and Cr, (**e**) line scan position of STEM-EDS, and the line scan results of (**f**) Al, (**g**) Cr and (**h**) O.

The TEM observation reveals the formation of an amorphous Al_2_O_3_ coating layer on the surface of the Cr_8_O_21_ particle (Figure 2a, taking the 12 ALD sample as an example) [29,30,31]. The HRTEM shows that the Al_2_O_3_ layer has a noncrystalline structure [29], while the Cr_8_O_21_ particle shows clear crystalline patterns. The thickness of the Al_2_O_3_ layer is approximately 4.13 nm. In the ALD process, the Al_2_O_3_ layer thickness can reach 1 nm per 3 cycles (The thickness calibration results by AFM are shown in Figure S3 and Figure S4). Therefore, the thickness of the Al_2_O_3_ layer of the 12 ALD cycles sample must be approximately 4 nm, in accordance with the TEM observation. The lattice fringe of the particle core is 0.387 nm, which corresponds to the (1 1 1) planes of Cr_8_O_21_. It is also shown that the crystal structure of Cr_8_O_21_ is not affected by the ALD coating process, which can be confirmed by XRD results of the Cr_8_O_21_ electrodes with different ALD cycles (Figure S5). EDS mapping of TEM (Figure 2b–d) clearly shows that the aluminum atoms are uniformly distributed on the surface of the Cr_8_O_21_ particle, indicating that the Al_2_O_3_ ALD membrane is uniformly coated on the surface of the Cr_8_O_21_ electrode. The line scan in Figure 2e–h indicates an aluminum-rich layer at the surface of the particle, which corresponds to the thickness of the Al_2_O_3_ ALD layer.

**Figure 3:**
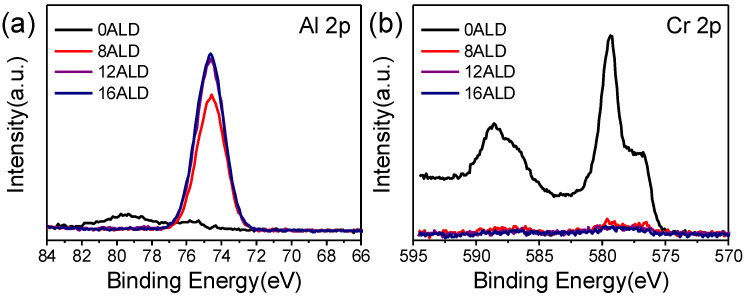
XPS spectra of (**a**) Al 2p, (**b**) Cr 2p with different coating cycle numbers (0, 8, 12, 16).

XPS was used to determine the composition of the ALD layer. Figure S6 shows the XPS spectra of the Cr_8_O_21_ electrode with different ALD cycles (0, 8, 12, 16), and Figure 3a displays the XPS peak of Al2p with different coating cycle numbers (0, 8, 12, 16). For the electrode without Al_2_O_3_ coating, the intensity of the Al2p peak is almost zero. The intensity of Al2p peaks increases with the increase in coating cycle numbers. However, when the coating cycle number reaches 12, the increase in Al2p peak intensity is no longer discernable [30,31,32,33,34]. It is well known that XPS can provide only an analysis depth of a few nanometers. Here, the thickness of Al_2_O_3_ ALD film is to be approximately 1 nm per 3 cycles. As 12 ALD coating was performed, the thickness value may achieve the limit of XPS detection depth. Therefore, even if the ALD cycles increase to 16, the intensity of the Al element is no longer significantly increased. The XPS spectra of Cr2p with different coating cycle numbers (0, 8, 12, 16) are shown in Figure 3b. When there is no ALD coating on the Cr_8_O_21_ electrode, the discernable XPS spectrum of Cr2p can be observed. When the Al_2_O_3_ ALD layer is coated on the electrode, the signal intensity of chromium decreases obviously (XPS spectra of Cr 2p with different coating cycle numbers (8, 12, 16) are shown in Figure S7). This is due to the fact that the thick Al_2_O_3_ layer on the electrode surface completely blocked the penetration of the X-ray, which corresponds to the variation of the signal intensity of the aluminum element.

**Figure 4:**
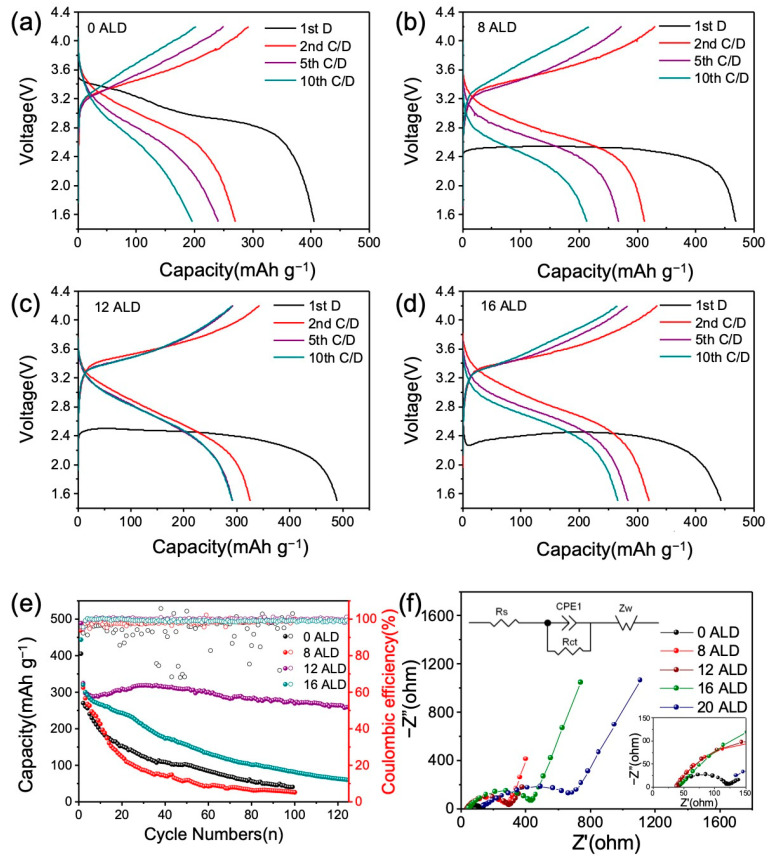
Charge/discharge curves of Cr_8_O_21_ all-solid-state cells with different ALD cycles at 60 °C: (**a**) without Al_2_O_3_ coating, (**b**) 8 ALD cycles, (**c**) 12 ALD cycles, (**d**) 16 ALD cycles; (**e**) Cycling performance (the left vertical axis) and CE (the right vertical axis) of the Cr_8_O_21_ all-solid-state cell with different ALD cycles (0, 8, 12, 16). (**f**) EIS profiles of the Cr_8_O_21_ all-solid-state cell with different ALD cycles (0, 8, 12, 16, 20) at 60 °C and the equivalent circuit model of EIS (inset).

To evaluate the effects of the ALD coating Al_2_O_3_, ASS cells with ALD-Al_2_O_3_-coated Cr_8_O_21_ cathodes, PEO-based polymer electrolyte membranes, and Li metal anodes were assembled and electrochemically tested. The electrochemical operation window of the PEO-based polymer electrolyte membranes was estimated to be 1.0–4.6 V, as shown in Figure S8. Therefore, the voltage range of the charging/discharging test was set to 1.5–4.2 V for the ASS cells. Considering that Cr_8_O_21_ has strong oxidizing property, which may degrade organic substances, the Al_2_O_3_ ALD coating on the electrode performed at least eight cycles (labeled 8ALD). Then, the samples with different thicknesses of ALD coating were obtained after 12 cycles (12ALD), 16 cycles (16ALD), and 20 cycles (20ALD). As shown in Figure 4a, the ASS cell with an uncoated ALD electrode displays a similar discharge profile with the conventional organic electrolyte cell (Figure S9). However, the ASS cell with an uncoated ALD electrode delivers a lower discharge capacity of 405 mAh g^−1^ compared with that of the organic electrolyte cell (450 mAh g^−1^). The decline of discharge capacity is attributed to the strong oxidizability of Cr_8_O_21_ material, which leads to oxidation of PEO electrolyte membrane during the rest at 60 °C for 30 min before testing. As the PEO membrane is oxidized, part chromium atoms in Cr_8_O_21_ are reduced to a lower valence state concurrently, which results in a decrease in discharge capacity. When the electrodes are coated by Al_2_O_3_ ALD, the discharge voltage decreases with the coating cycle. This phenomenon is ascribed to the Al_2_O_3_ ALD that increases the resistance for both electron and ion conduction [35,36,37], which serves as a barrier to ion and electron mobility in the charge/discharge process. For this reason, increasing coating thickness leads to gradually increasing polarization resistance [38,39,40]. The thickness of the ALD coating produces a completely opposite effect on the oxidation resistance and the polarization effect, resulting in a tendency for the discharge capacity to increase first and then decreasing. The discharge capacity of Cr_8_O_21_ gradually increases from 470 to 490 mAh g^−1^ when the coating cycles increase from 8 to 12 ALD. After that, the discharge capacity decreases with the increase in coating cycles. When the electrode was coated by 20 ALD, the discharge capacity was close to zero. The change of capacity can be explained by the synergy of three factors, including temperature, coating cycles, and the reaction of the electrode with the PEO membrane. The discharge capacity of the cell at 60 °C is higher than that at room temperature due to that elevation of temperature not only facilitates the migration of Li ions but also promotes the chemical reaction in the cell. On the one hand, ALD coating can inhibit the reduction in the Cr_8_O_21_ electrode and increase the polarization resistance. When the ALD cycles are less than 12, reduction in the Cr_8_O_21_ electrode and oxidation of the electrolyte membrane cannot be completely suppressed. The redox reaction between the electrode and the membrane reduces the discharge capacity. When the ALD coating cycle reaches 12, the redox reaction between the electrode and the membrane is completely overwhelmed, which leads to the maximum discharge capacity of 490 mAh g^−1^. As the ALD cycle is greater than 12, although the redox reaction can be suppressed, the increase in polarization reduces the discharge capacity. When the coating cycle is 20, the electrode delivers no discharge capacity at all, indicating that the thicker layer of Al_2_O_3_ completely impedes the diffusion of lithium ions.

Figure 4e displays the cycling performance of the Cr_8_O_21_ all-solid-state cell with different ALD cycles. When the coating cycle is less than 12, the discharge capacity rapidly declines to approximately 200 mAh g^−1^ after 10 cycles. Up cycling to 100 cycles, the discharge capacity is 40 mAh g^−1^ and 26 mAh g^−1^ for the 0ALD and 8ALD cells, respectively. The 8ALD cell has a worse cycling performance compared with 0ALD, which is mainly attributed to the coincidence of redox reaction and polarization effect. It is worth noting that the discharge capacity becomes stable from the 5th charge/discharge cycle when the ALD coating cycle is 12. It delivers a capacity of 260 mAh g^−1^ for the 125th cycle at 0.1 C with a discharge-specific energy of 630 Wh kg^−1^. However, 16 ALD is found to deteriorate the cycling performance, although it can prevent the redox reaction occurrence completely [27,28,40]. Coulombic efficiency (CE) can be used to estimate the cycling life of LIB. Figure 4e also shows that the CE of the cell with 12 ALD cycles layer is higher than that of the others, which is consistent with the cyclic performance. It was reported that ALD Al_2_O_3_ is not thermodynamically stable at low potentials. A stable Li-Al-O phase with high lithium-ion conductivity first forms after lithiation at low potentials, which is beneficial to the rapid transport of lithium ions [41,42,43,44,45,46,47,48]. Then the superfluous lithium ions can flow through the Li-Al-O phase to the adjacent active materials to participate in the charge/discharge reactions. A recent study also suggested that the Li-Al-O phase can be generated directly on the surface of a lithium electrode [49]. This may explain the experimental results when the ALD cycles are less than 12. A thick layer of Al_2_O_3_ would inhibit the diffusion of lithium ions, thereby deteriorating the battery performance [43,50,51,52]. Furthermore, it also increases the distance of ion transport. These negative effects may induce the deterioration of cycling performance with coating cycles of more than 12 ALD.

The effect of resistance on discharge performance was further verified by EIS tests, as shown in Figure 4f and Table 1. The equivalent circuit model of EIS analysis is displayed in Figure 4f. All EIS curves consist of a semicircle in a high-frequency range and an inclined straight line in a low-frequency range. It can be seen that the Al_2_O_3_ coating layer does not affect the electrochemical reaction process, which is the same as the previous report [53]. The depressed semicircle in the high-frequency region is assigned to the charge transfer process (Rct), and the inclined line in the low-frequency region is related to the Li-ion diffusion resistance (Zw). Figure 4f and Table 1 indicate that when the ALD coating cycles were less than 20, all the Rs (series ohmic resistances) were approximately 42 Ω. This shows that thin ALD coating has little effect on Rs. The slight change in Rs may be caused by the difference in the preparation process of the coin cells. For the electrodes with ALD coating cycles of more than 16, increasing the thickness of ALD coating obviously leads to the increase in Rs. The Rct increases obviously with the increase in the number of ALD coating cycles. For the electrode without ALD coating, the Rct is 79.2 Ω. When eight cycles of ALD were coated on the electrode, Rct increased to 252.9 Ω. Continually increases the ALD coating cycles to 20, Rct reaches an amazing value of 660.9 Ω. The large resistance of the 20-ALD-coated electrode prevents it from releasing any capacity. The result is consistent with the variation of the first discharge plateau. Table 1 and Figure S10 also show that the 0 ALD electrode has minimum Warburg coefficient (σ) [54], on the other hand, of all ALD-coated electrodes, 12 ALD electrode has a minimum Warburg coefficient (σ), which is consistent with the results of electrochemical performance tests.

**Figure 5:**
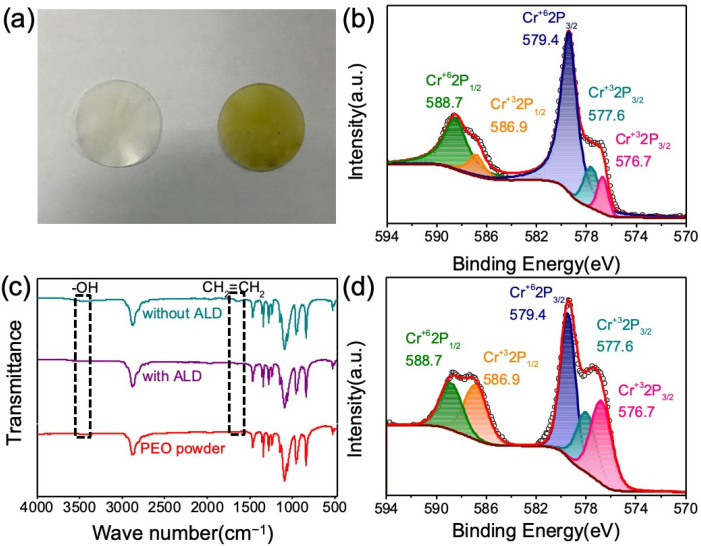
(**a**) Macrographs of PEO membranes contacted with Cr_8_O_21_ electrodes (with 12 ALD (left) and without ALD (right)) at 60 °C for 7 days; (**b**) Cr 2p XPS spectra of pristine Cr_8_O_21_ electrodes; (**c**) FTIR spectra of three types of PEO (without ALD, with ALD and PEO powder); (**d**) Cr 2p XPS spectra of the Cr_8_O_21_ electrode contacting with PEO membranes.

To further explicate the effect of ALD coating on the cycling performance of ASSB, the PEO membranes at different states were subjected to FTIR analysis, and the associated Cr_8_O_21_ electrodes were characterized by XPS analysis. Figure 5a shows the macrographs of the PEO electrolyte membranes directly contacted with the Cr_8_O_21_ electrode or the ALD-coated electrode after keeping at 60 °C for a week. The color of the membrane tightly bonded to the electrode with ALD coating remains unchanged, while the color of the membrane tightly bound to the electrode without ALD coating turned yellow. This might be attributed to the degradation of PEO by Cr_8_O_21_ oxidation. Figure 5c illustrates the FTIR spectra of the PEO membranes contacting Cr_8_O_21_ with and without ALD coating and pure PEO powder. It is obvious that the FTIR patterns are similar. Figure S11 and Figure S12 are partially enlarged spectra in the range of 1560 to 1720 cm^−1^ and 3200 to 3600 cm^−1^, respectively. It is found that there are two weak absorption peaks at 3425 and 1640 cm^−1^ for the PEO membrane without ALD. The two peaks correspond to the vibration of the hydroxyl group and the carbon double bond, which can be ascribed to oxidative degradation reaction of PEO. This is consistent with the results reported in the literature [55,56]. In contrast, there are no corresponding vibration peaks for the membrane with ALD and pure PEO powder. Therefore, the ALD coating can effectively prevent the PEO from being oxidized. Figure 5b shows the XPS spectrum (Cr 2p) of the pristine Cr_8_O_21_ electrodes, in which the intensity ratio of Cr^+6^/Cr^+3^ peaks is higher than that of the Cr_8_O_21_ electrode contacting with PEO membranes, as shown in Figure 5d.

On the basis of the above discussion, the roles of the ALD Al_2_O_3_ layer in improving the performance of the all-solid-state batteries are summarized as follows: (i) The dense Al_2_O_3_ layer can physically insulate the electrode (Cr_8_O_21_) from direct contact with the polymer electrolyte membrane (PEO), which avoids side effects between Cr_8_O_21_ and PEO. (ii) Although Al_2_O_3_ itself is an insulator of electrons and ions, a suitable thickness (it is approximately 4 nm in this study) of the Al_2_O_3_ layer could form a stable Li-Al-O phase after lithiation, which is beneficial to the rapid transport of lithium ions. (iii) Li-Al-O has a stable chemical property, which can improve the interfacial stability between the solid electrolyte and the electrode (Figure S13). (iv) The Al_2_O_3_ ALD coating can not only mechanically restrict the pulverization of the active material but also prevent the electrode materials from peeling off the current collector. It eventually improves the cycling stability of the electrode.

## 4. Conclusions

In summary, the ALD technique was applied to improve the electrochemical performance of the Cr_8_O_21_/Li all-solid-state batteries. The dense Al_2_O_3_ layer was evenly coated on the surface of the Cr_8_O_21_ electrode. The Al_2_O_3_ layer prevents the direct contact between the Cr_8_O_21_ electrode and the PEO film; therefore, the oxidation of the PEO electrolyte membrane by Cr_8_O_21_ is depressed. Meanwhile, the thin Al_2_O_3_ layer allows lithium ions to pass through and ensures the electrochemical reactions. The electrochemical tests indicated that the 12-cycle ALD-Al_2_O_3_-coated electrode delivers a high capacity of 260 mAh g^−1^ after 125 cycles at 0.1 C with a discharge-specific energy of 630 Wh kg^−1^. The ALD technique may also be used in other battery systems for stabilizing the interface between the electrode and the solid electrolyte.

## Figures and Tables

**Table 1:** Impedance parameters of Cr_8_O_21_ all-solid-state cell with different ALD cycles (0, 8, 12, 16, 20).

	0 ALD	8 ALD	12 ALD	16 ALD	20 ALD
Rs (Ω)	42.8	39.7	39.3	46.3	126.4
Rct (Ω)	79.2	252.9	259.7	405.4	660.9
σ	17.84	130.84	95.73	333.29	1148.46

## Data Availability

Not applicable.

## Supplementary Materials

The following are available online at https://www.mdpi.com/article/10.3390/ma14185380/s1, Figure S1: SEM images of Cr_8_O_21_ electrodes with different ALD cycles: (a) 0 ALD; (b) 8 ALD; (c) 12 ALD; (d) 16 ALD. Figure S2: Al element content of Cr_8_O_21_ electrodes with different ALD cycles (0, 8, 12, 16). Figure S3: The thickness test result of 100-ALD-cycles Al_2_O_3_ coated on a silicon plate. Figure S4: The AFM results of 100-ALD-cycles Al_2_O_3_ coated on silicon plate: (a) line position; (b) the thickness test result of 100 ALD cycles Al_2_O_3_. Figure S5: The XRD patterns of Cr_8_O_21_ electrodes with different ALD cycles. Figure S6: XPS spectra of Cr_8_O_21_ electrodes. Figure S7: XPS spectra of Cr 2p with different coating cycle numbers (8, 12, 16). Figure S8: CV curves of PEO members at a scan rate of 0.1 mV s^−1^ (60 °C). Figure S9: Galvanostatic charge/discharge profiles of Cr_8_O_21_ electrode with liquid electrolyte (1 M LiPF6 EC/DMC). Figure S10: The linear fits of the relationship between Z′ and ω^−0.5^ of Cr_8_O_21_ electrode with different ALD cycles (0, 8, 12, 16, 20). Figure S11: The partially enlarged spectra in the range of 1560 cm^−1^ to 1720 cm^−1^. Figure S12: The partially enlarged spectra in the range of 3200 cm^−1^ to 3600 cm^−1^. Figure S13: Electrochemical polarization voltage of Cr_8_O_21_ electrode with different ALD cycles (0, 12).

## References

[1] Goodenough J.B. (2018). How we made the Li-ion rechargeable battery. Nat. Electron..

[2] Nitta N., Wu F., Lee J.T., Yushin G. (2015). Li-ion battery materials: Present and future. Mater. Today.

[3] Whittingham M.S. (2004). Lithium batteries and cathode materials. Chem. Rev..

[4] Wang N., Chen B., Qin K., Liu E., Shi C., He C., Zhao N. (2019). Rational design of Co_9_S_8_/CoO heterostructures with well-defined interfaces for lithium sulfur batteries: A study of synergistic adsorption-electrocatalysis function. Nano Energy.

[5] Masset P.J., Guidotti R.A. (2008). Thermal activated (“thermal”) battery technology. J. Power Sour..

[6] Xiao A.W., Lee H.J., Capone I., Robertson A., Wi T.U., Fawdon J., Wheeler S., Lee H.W., Grobert N., Pasta M. (2020). Understanding the conversion mechanism and performance of monodisperse FeF_2_ nanocrystal cathodes. Nat. Mater..

[7] Cabana J., Monconduit L., Larcher D., Palacin M.R. (2010). Beyond intercalation-based Li-ion batteries: The state of the art and challenges of electrode materials reacting through conversion reactions. Adv. Mater..

[8] Mei J., Liao T., Kou L., Sun Z. (2017). Two-dimensional metal oxide nanomaterials for next-generation rechargeable batteries. Adv. Mater..

[9] Liu J., Wang Z., Li H., Huang X. (2006). Synthesis and characterization of Cr_8_O_21_ as cathode material for rechargeable lithium batteries. Solid State Ion..

[10] Guo Y., Li H., Zhai T. (2017). Reviving lithium-metal anodes for next-generation high-energy batteries. Adv. Mater..

[11] Lin D., Liu Y., Cui Y. (2017). Reviving the lithium metal anode for high-energy batteries. Nat. Nanotechnol..

[12] Cheng X.B., Zhang R., Zhao C.Z., Zhang Q. (2017). Toward safe lithium metal anode in rechargeable batteries: A review. Chem. Rev..

[13] Feng G.X., Li L.F., Liu J.Y., Liu N., Li H., Yang X.Q., Huang X.J., Chen L.Q., Nam K.W., Yoon W.S. (2009). Enhanced electrochemical lithium storage activity of LiCrO_2_ by size effect. J. Mater. Chem..

[14] Zhang R., Wen S., Wang N., Qin K., Liu E., Shi C., Zhao N. (2018). N-doped graphene modified 3D porous Cu current collector toward microscale homogeneous Li deposition for Li metal anodes. Adv. Energy Mater..

[15] Cheng X.-B., Zhao C.-Z., Yao Y.-X., Liu H., Zhang Q. (2019). Recent advances in energy chemistry between solid-state electrolyte and safe lithium-metal anodes. Chem.

[16] Xia S., Wu X., Zhang Z., Cui Y., Liu W. (2019). Practical challenges and future perspectives of all-solid-state lithium-metal batteries. Chem.

[17] Zhao Y., Zheng K., Sun X. (2018). Addressing interfacial issues in liquid-based and solid-state batteries by atomic and molecular layer deposition. Joule.

[18] Shen Y., Zhang Y., Han S., Wang J., Peng Z., Chen L. (2018). Unlocking the energy capabilities of lithium metal electrode with solid-state electrolytes. Joule.

[19] Long L., Wang S., Xiao M., Meng Y. (2016). Polymer electrolytes for lithium polymer batteries. J. Mater. Chem. A.

[20] Sun C., Liu J., Gong Y., Wilkinson D.P., Zhang J. (2017). Recent advances in all-solid-state rechargeable lithium batteries. Nano Energy.

[21] Zhang Y., Lai J., Gong Y., Hu Y., Liu J., Sun C., Wang Z.L. (2016). A safe high-performance all-solid-state lithium-vanadium battery with a freestanding V_2_O_5_ nanowire composite paper cathode. ACS Appl. Mater. Interfaces.

[22] Shim J., Kim D.G., Kim H.J., Lee J.H., Lee J.C. (2015). Polymer composite electrolytes having core-shell silica fillers with anion-trapping boron moiety in the shell layer for all-solid-state lithium-ion batteries. ACS Appl. Mater. Interfaces.

[23] Han F., Yue J., Chen C., Zhao N., Fan X., Ma Z., Gao T., Wang F., Guo X., Wang C. (2018). Interphase engineering enabled all-ceramic lithium battery. Joule.

[24] Luntz A.C., Voss J., Reuter K. (2015). Interfacial challenges in solid-state Li ion batteries. J. Phys. Chem. Lett..

[25] Ohta N., Takada K., Zhang L., Ma R., Osada M., Sasaki T. (2006). Enhancement of the high-rate capability of solid-state lithium batteries by nanoscale interfacial modification. Adv. Mater..

[26] Norby P. (1991). The crystal structure of Cr_8_O_21_ determined from powder diffraction data thermal transformation and magnetic properties of a chromium-chromate-tetrachromate. J. Solid State Chem..

[27] Guan D., Jeevarajan J.A., Wang Y. (2011). Enhanced cycleability of LiMn_2_O_4_ cathodes by atomic layer deposition of nanosized-thin Al_2_O_3_ coatings. Nanoscale.

[28] Lee J.T., Wang F.M., Cheng C.S., Li C.C., Lin C.H. (2010). Low-temperature atomic layer deposited Al_2_O_3_ thin film on layer structure cathode for enhanced cycleability in lithium-ion batteries. Electrochim. Acta.

[29] Kosova N.V., Devyatkina E.T. (2007). Comparative study of LiCoO_2_ surface modified with different oxides. J. Power Sour..

[30] Riley L.A., Van Atta S., Cavanagh A.S., Yan Y., George S.M., Liu P., Dillon A.C., Lee S.-H. (2011). Electrochemical effects of ALD surface modification on combustion synthesized LiNi_1/3_Mn_1/3_Co_1/3_O_2_ as a layered-cathode material. J. Power Sour..

[31] Zhou A., Liu Q., Wang Y., Wang W., Yao X., Hu W., Zhang L., Yu X., Li J., Li H. (2017). Al_2_O_3_ surface coating on LiCoO_2_ through a facile and scalable wet-chemical method towards high-energy cathode materials withstanding high cutoff voltages. J. Mater. Chem. A.

[32] Yu M., Yuan W., Li C., Hong J.-D., Shi G. (2014). Performance enhancement of a graphene–sulfur composite as a lithium–sulfur battery electrode by coating with an ultrathin Al_2_O_3_ film via atomic layer deposition. J. Mater. Chem. A.

[33] Li X., Liu J., Wang B., Banis M.N., Xiao B., Li R., Sham T.-K., Sun X. (2014). Nanoscale stabilization of Li–sulfur batteries by atomic layer deposited Al_2_O_3_. RSC Adv..

[34] Bettge M., Li Y., Sankaran B., Rago N.D., Spila T., Haasch R.T., Petrov I., Abraham D.P. (2013). Improving high-capacity Li_1.2_Ni_0.15_Mn_0.55_Co_0.1_O_2_-based lithium-ion cells by modifiying the positive electrode with alumina. J. Power Sour..

[35] Lu W., Liang L., Sun X., Sun X., Wu C., Hou L., Sun J., Yuan C. (2017). Recent progresses and development of advanced atomic layer deposition towards high-performance Li-ion batteries. Nanomaterials.

[36] Guan C., Wang J. (2016). Recent development of advanced electrode materials by atomic layer deposition for electrochemical energy storage. Adv. Sci..

[37] Jung Y.S., Cavanagh A.S., Riley L.A., Kang S.H., Dillon A.C., Groner M.D., George S.M., Lee S.H. (2010). Ultrathin direct atomic layer deposition on composite electrodes for highly durable and safe Li-ion batteries. Adv. Mater..

[38] Kim J.W., Kim D.H., Oh D.Y., Lee H., Kim J.H., Lee J.H., Jung Y.S. (2015). Surface chemistry of LiNi_0.5_Mn_1.5_O_4_ particles coated by Al_2_O_3_ using atomic layer deposition for lithium-ion batteries. J. Power Sour..

[39] Seok Jung Y., Cavanagh A.S., Yan Y., George S.M., Manthiram A. (2011). Effects of atomic layer deposition of Al_2_O_3_ on the Li[Li_0.20_Mn_0.54_Ni_0.13_Co_0.13_]O_2_ cathode for lithium-ion batteries. J. Electrochem. Soc..

[40] Su Y., Cui S., Zhuo Z., Yang W., Wang X., Pan F. (2015). Enhancing the high-voltage cycling performance of LiNi_0.5_Mn_0.3_Co_0.2_O_2_ by retarding its interfacial reaction with an electrolyte by atomic-layer-deposited Al_2_O_3_. ACS Appl. Mater. Interfaces.

[41] Xiao X., Lu P., Ahn D. (2011). Ultrathin multifunctional oxide coatings for lithium ion batteries. Adv. Mater..

[42] Kazyak E., Wood K.N., Dasgupta N.P. (2015). Improved cycle life and stability of lithium metal anodes through ultrathin atomic layer deposition surface treatments. Chem. Mater..

[43] Wang L., Zhang L., Wang Q., Li W., Wu B., Jia W., Wang Y., Li J., Li H. (2018). Long lifespan lithium metal anodes enabled by Al_2_O_3_ sputter coating. Energy Storage Mater..

[44] Jung S.C., Han Y.-K. (2013). How do Li atoms pass through the Al_2_O_3_ coating layer during lithiation in Li-ion batteries?. J. Phys. Chem. Lett..

[45] Han X., Gong Y., Fu K.K., He X., Hitz G.T., Dai J., Pearse A., Liu B., Wang H., Rubloff G. (2017). Negating interfacial impedance in garnet-based solid-state Li metal batteries. Nat. Mater..

[46] Hao S., Wolverton C. (2013). Lithium transport in amorphous Al_2_O_3_ and AlF_3_ for discovery of battery coatings. J. Phys. Chem. C.

[47] Jung S.C., Kim H.J., Choi J.W., Han Y.K. (2014). Sodium ion diffusion in Al_2_O_3_: A distinct perspective compared with lithium ion diffusion. Nano Lett..

[48] Liu Y., Hudak N.S., Huber D.L., Limmer S.J., Sullivan J.P., Huang J.Y. (2011). In situ transmission electron microscopy observation of pulverization of aluminum nanowires and evolution of the thin surface Al_2_O_3_ layers during lithiation-delithiation cycles. Nano Lett..

[49] Xie M., Lin X., Huang Z., Li Y., Zhong Y., Cheng Z., Yuan L., Shen Y., Lu X., Zhai T. (2019). A Li–Al–O solid-state electrolyte with high ionic conductivity and good capability to protect Li anode. Adv. Funct. Mater..

[50] Han X., Liu Y., Jia Z., Chen Y.C., Wan J., Weadock N., Gaskell K.J., Li T., Hu L. (2014). Atomic-layer-deposition oxide nanoglue for sodium ion batteries. Nano Lett..

[51] Li Y., Sun Y., Xu G., Lu Y., Zhang S., Xue L., Jur J.S., Zhang X. (2014). Tuning electrochemical performance of Si-based anodes for lithium-ion batteries by employing atomic layer deposition alumina coating. J. Mater. Chem. A.

[52] Lipson A.L., Puntambekar K., Comstock D.J., Meng X., Geier M.L., Elam J.W., Hersam M.C. (2014). Nanoscale investigation of solid electrolyte interphase inhibition on li-ion battery mno electrodes via atomic layer deposition of Al_2_O_3_. Chem. Mater..

[53] Jing H.K., Kong L.L., Liu S., Li G.R., Gao X.P. (2015). Protected lithium anode with porous Al_2_O_3_ layer for lithium–sulfur battery. J. Mater. Chem. A..

[54] Chen B., Lu H., Zhou J., Ye C., Shi C., Zhao N., Qiao S.-Z. (2018). Porous MoS_2_/carbon spheres anchored on 3D interconnected multiwall carbon nanotube networks for ultrafast Na storage. Adv. Energy Mater..

[55] Jones G.K., McGhie A.R., Farrington G.C. (1991). Studies of the stability of poly(ethylene oxide) and PEO-based solid electrolytes using thermogravimetry-mass spectrometr. Macromolecules.

[56] Han S., Kim C., Kwon D. (1995). Thermal degradation of poly(ethyleneglycol). Polym. Degrad. Stab..

