# Supplementary material for: Enhanced Cyclability of Cr8O21 Cathode for PEO-Based All-Solid-State Lithium-Ion Batteries by Atomic Layer Deposition of Al2O3
Source: Materials (Basel). 2021 Sep 17;14(18):5380. doi: 10.3390/ma14185380 (PMC8468447; doi:10.3390/ma14185380)
Supplement: Supplementary file 1 [file materials-14-05380-s001.zip › materials-1359895-supplementary.pdf]

Supplementary Materials

Enhanced Cyclability of Cr_8O_{21} Cathode for PEO-Based All-Solid-State Lithium Ion Batteries by Atomic Layer Deposition of Al_2O_3

Haichang Zhang [1,2], Zhibin Xu [2], Bin Shi [2], Fei Ding [2,*], Xingjiang Liu [2], Hongzhao Wu [3], Chunsheng Shi [1,*] and Naiqin Zhao [1]

[1] School of Materials Science and Engineering, Tianjin Key Laboratory of Composite and Functional Materials, Tianjin University, Tianjin 300350, China; hchzhang@tju.edu.cn (H.Z.); nqzhao@tju.edu.cn (N.Z.)
[2] Science and Technology on Power Sources Laboratory, Tianjin Institute of Power Sources, Tianjin 300384, China; beyond_x@163.com (Z.X.); daiye101@mail.nankai.edu.cn (B.S.); xjliu@nklps.org (X.L.)
[3] School of Automotive Engineering, Tianjin Vocational Institute, Tianjin 300410, China; wuhongzhao9059@163.com
* Correspondence: fding@nklps.org (F.D.); csshi@tju.edu.cn (C.S.)

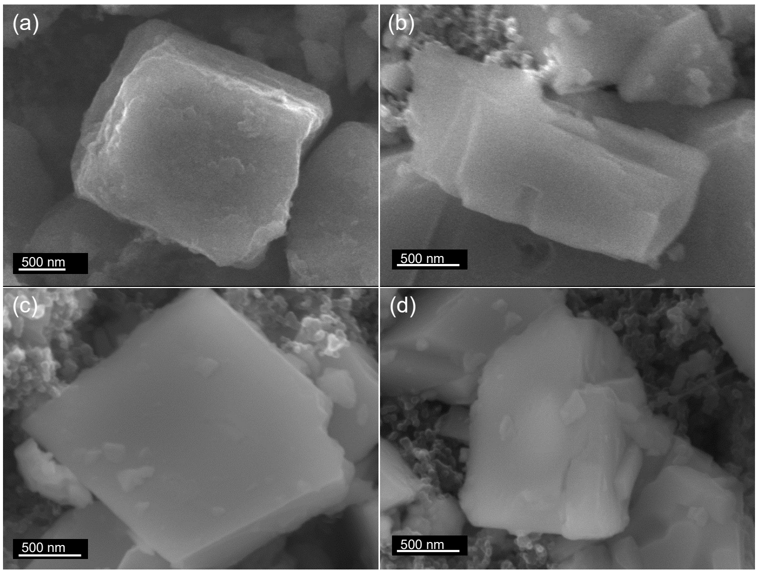

Figure S1. SEM images of Cr_8O_{21} electrodes with different ALD cycles: (**a**) 0 ALD; (**b**) 8 ALD; (**c**) 12 ALD; (d) 16 ALD.

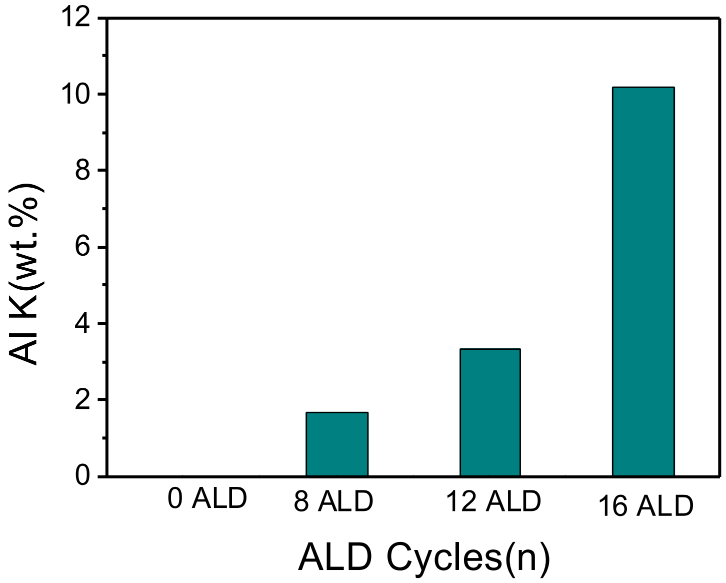

Figure S2. Al element content of Cr_8O_{21} electrodes with different ALD cycles (0, 8, 12, 16).

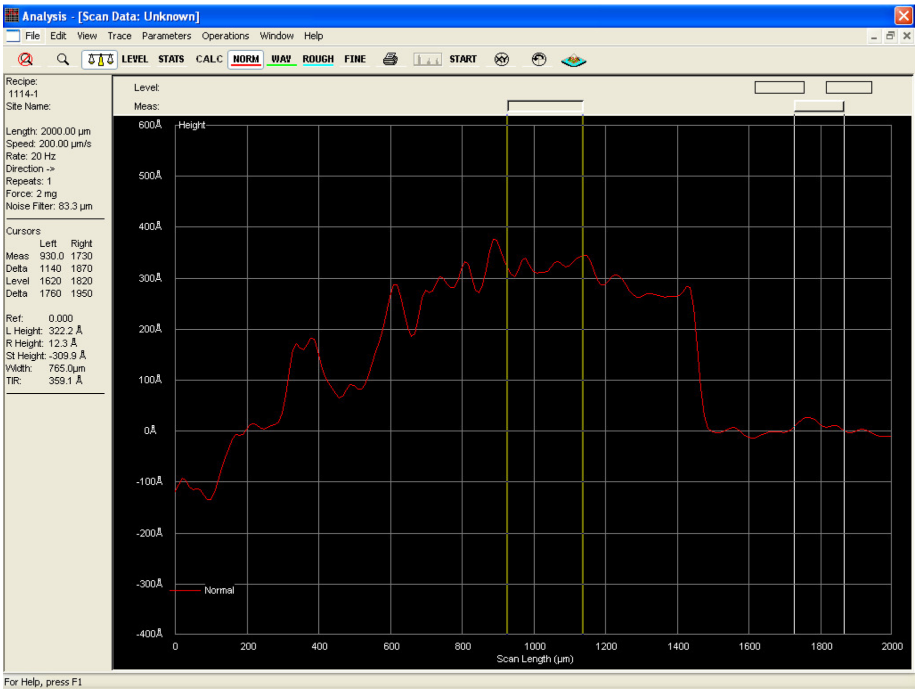

Figure S3. The thickness test result of 100 ALD cycles Al_2O_3 coated on silicon plate. (The thickness of 100 ALD cycles is about 33nm. So, the thickness of a ALD cycle is about 0.33nm).

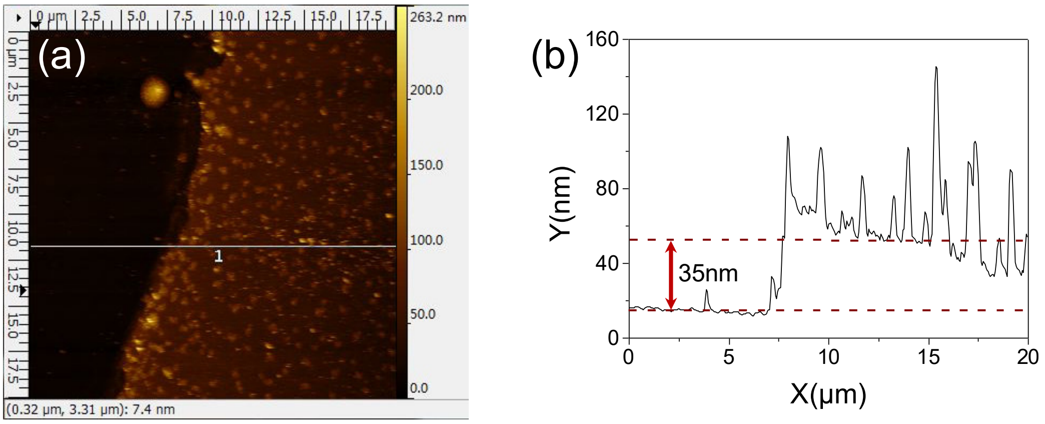

Figure S4. The AFM results of 100 ALD cycles Al_2O_3 coated on silicon plate:(**a**) line position; (**b**) the thickness test result of 100 ALD cycles Al_2O_3.

(In order to calibrate the deposition rate of Al_2O_3 ALD process, half of the silicon plate was glued with a PI tape, while the other half was exposed to ALD. After deposition by ALD, the PI tape was removed. The deposition rate was determined by dividing the measured height difference between the two halves by the number of deposition cycles. The deposition rate is related to the surface activity. The edge of the PI tape was rougher and had higher activity, so the deposition rate near the edge of the PI tape was larger and the thickness was larger, while the thickness away from the tape was smaller.)

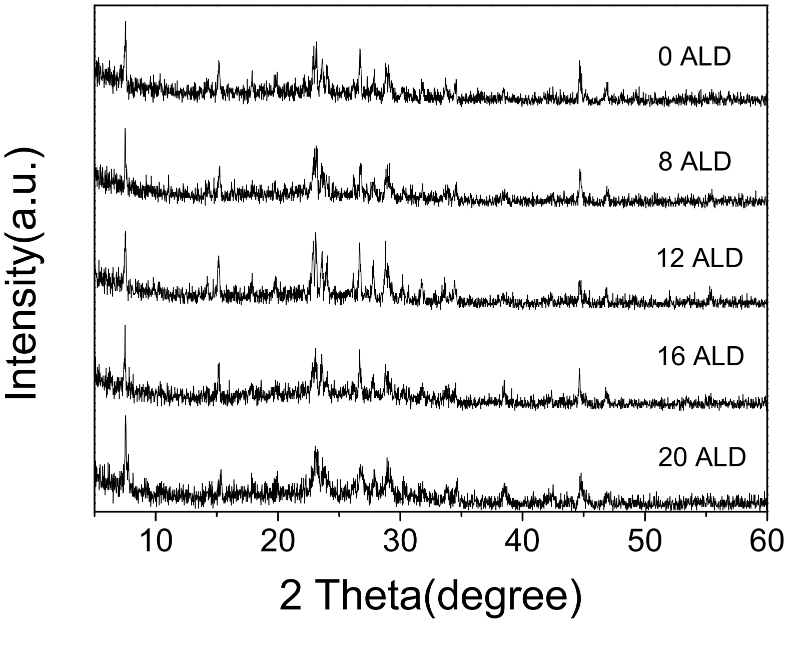

Figure S5. The XRD patterns of Cr_8O_{21} electrodes with different ALD cycles.

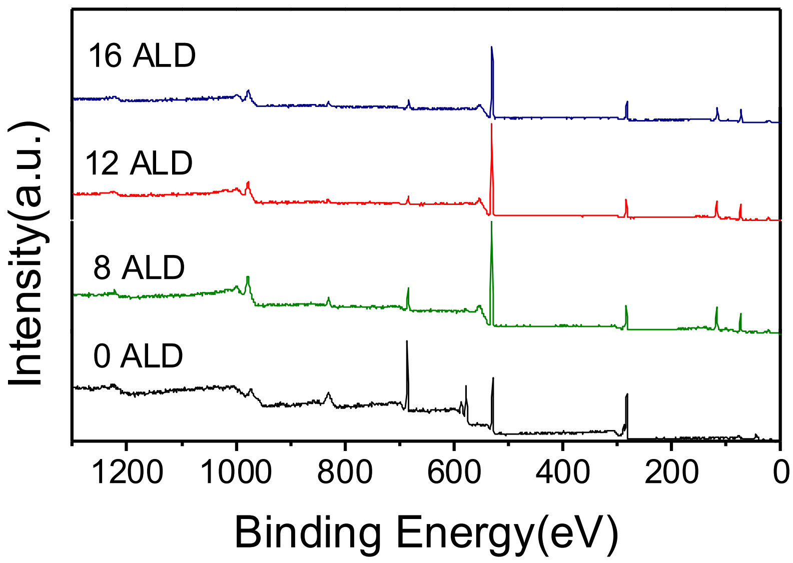

Figure S6. XPS spectra of Cr_8O_{21} electrodes.

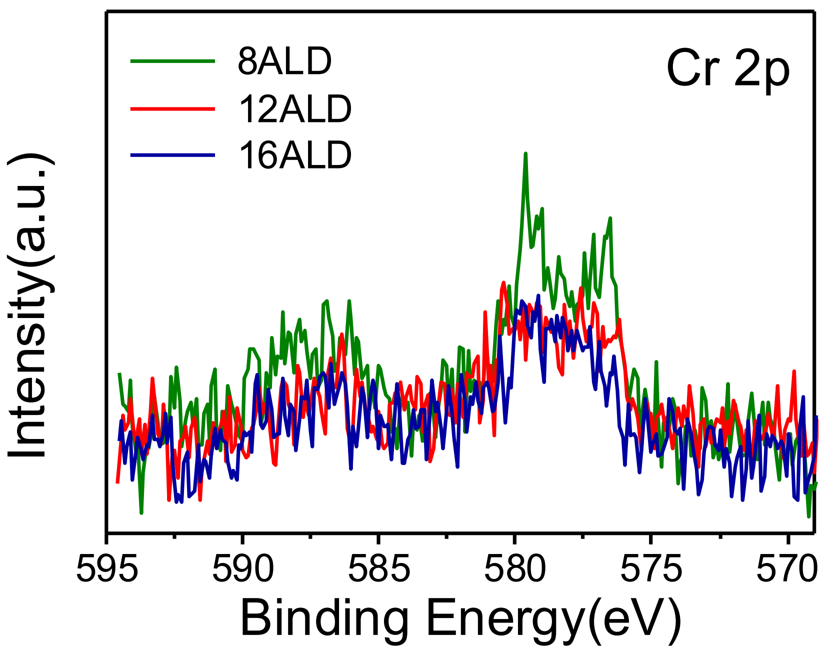

Figure S7. XPS spectra of Cr 2p with different coating cycle numbers (8, 12, 16).

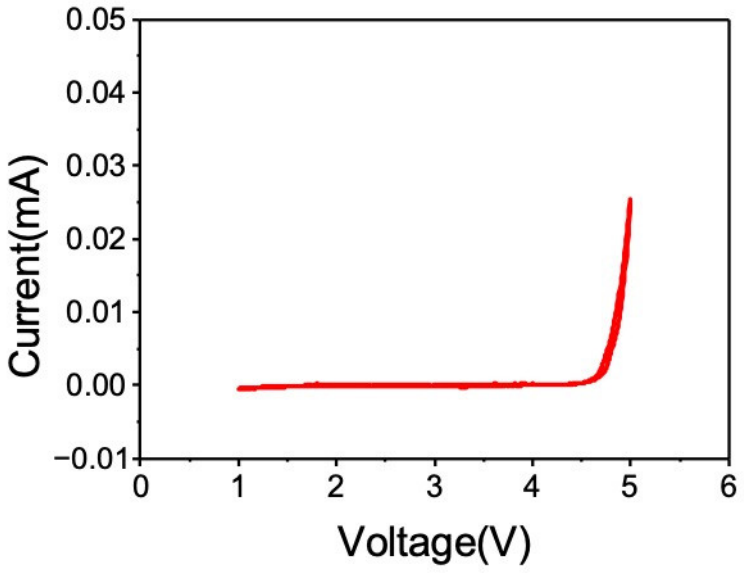

Figure S8. CV curves of PEO member at scan rate of 0.1mV s^{-1} (60°C).

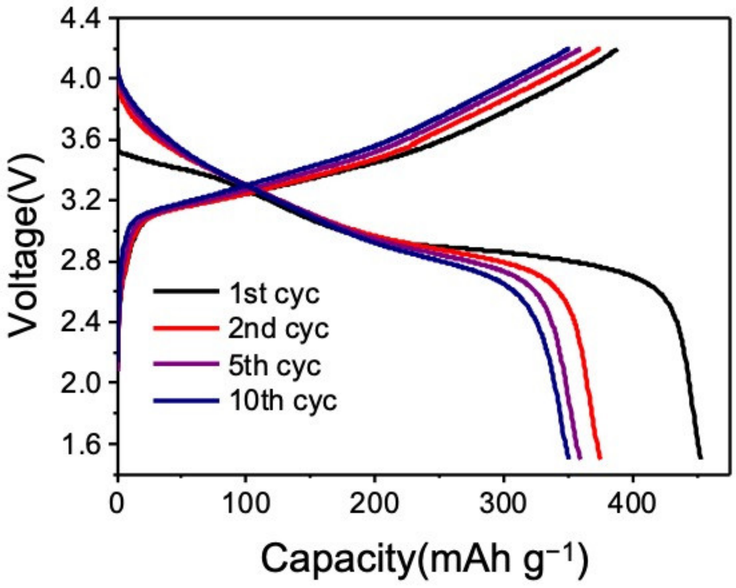

Figure S9. Galvanostatic charge/discharge profiles of Cr_8O_{21} electrode with liquid electrolyte (1M $LiPF_6$ EC/DMC).

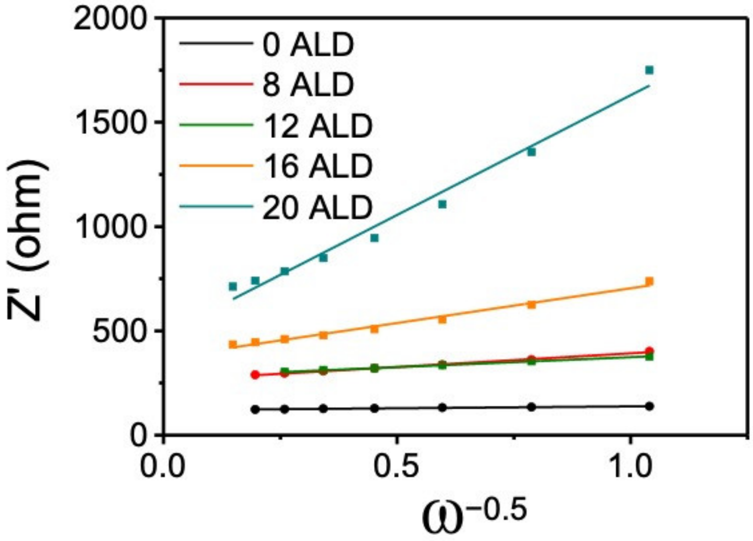

Figure S10. The linear fits of the relationship between Z′ and $\omega^{-0.5}$ of Cr_8O_{21} electrode with different ALD cycles (0, 8, 12, 16, 20).

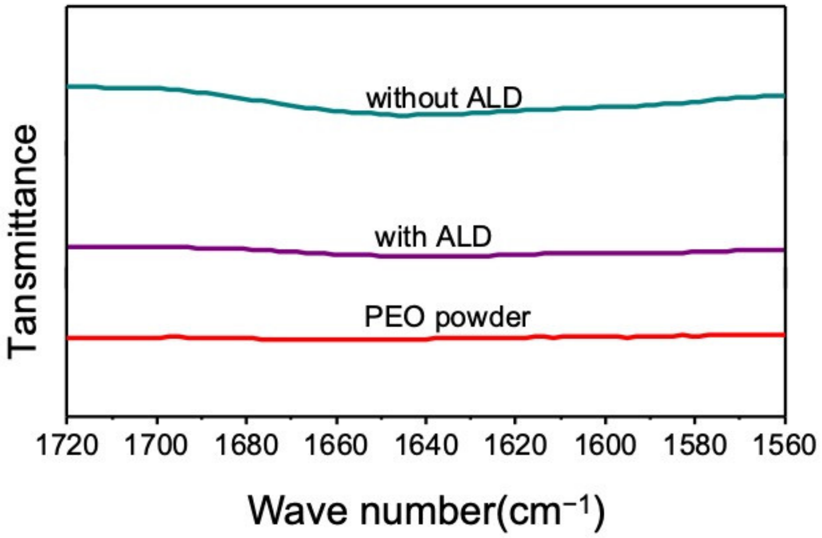

Figure S11. The partial enlarged spectra in the range of 1560 cm^{-1} to 1720 cm^{-1}.

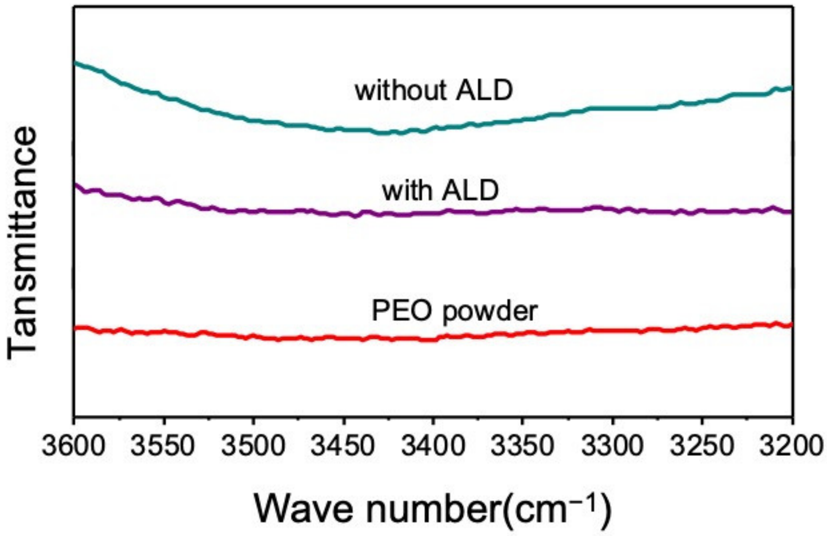

Figure S12. The partial enlarged spectra in the range of 3200 cm^{-1} to 3600 cm^{-1}.

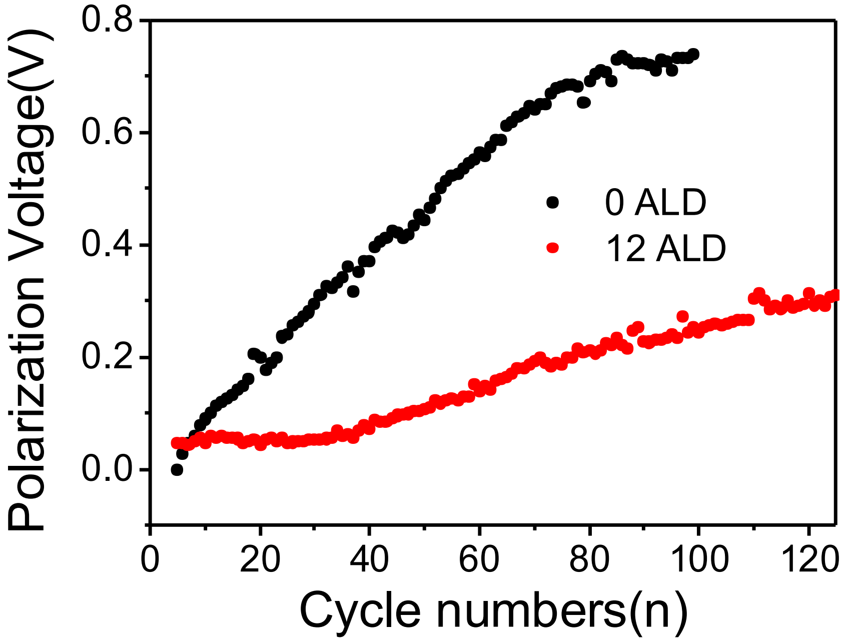

Figure S13. Electrochemical polarization voltage of Cr_8O_{21} electrode with different ALD cycles (0, 12).

Considering the irreversibility of the reaction of Cr_8O_{21} and the activation of solid-state battery, the polarization voltage be calculated from the fifth cycle.
